# Conservative management of complete fetal expulsion into the abdominal cavity after silent uterine rupture - case report

**DOI:** 10.1186/s12884-023-05812-1

**Published:** 2023-07-07

**Authors:** Lukas Hruban, Anna Jouzova, Petr Janku, Vit Weinberger, Dagmar Seidlova, Tomas Juren, Jan Senkyrik, Jana Kadlecova, Jitka Hausnerova, Eva Jandakova

**Affiliations:** 1 Department of Obstetrics and Gynecology, University Hospital Brno and Medical Faculty, Masaryk University, Jihlavská 20, Brno, 625 00 Czech Republic; 2 Department of Health Sciences, Faculty of Medicine, Masaryk University, Brno, Czech Republic; 3 Department of Anesthesiology and Intensive Care, University Hospital Brno and Medical Faculty, Masaryk University, Brno, Czech Republic; 4 Department of Neonatology, University Hospital Brno and Medical Faculty, Masaryk University, Brno, Czech Republic; 5 Department of Pediatric Radiology, University Hospital Brno and Medical Faculty, Masaryk University, Brno, Czech Republic; 6 Department of Pathology, University Hospital Brno and Medical Faculty, Masaryk University, Brno, Czech Republic

**Keywords:** Silent uterine rupture, Abdominal pregnancy, Fetal expulsion, Uterine scar

## Abstract

**Background:**

Clinically silent uterine rupture with complete fetal expulsion into the abdominal cavity is an extremely rare complication. Diagnosis can be difficult and the risk to the mother and fetus is high. Conservative management has been described only in a few cases of partial expulsion of the fetus so far.

**Case presentation:**

We present a case of 43-year-old tercigravida with a history of previous laparotomic myomectomy and subsequent cesarean section. The subsequent pregnancy was complicated by uterine wall loosening and rupture at the site of the previous uterine scar after myomectomy and complete fetal expulsion into the abdominal cavity. The diagnosis was made at 24 + 6 weeks of gestation. Considering the absence of clinical symptomatology and the good condition of the fetus, a conservative approach was chosen with intensive monitoring of the maternal and fetal conditions. The pregnancy ended by elective cesarean section and hysterectomy at 28 + 0 weeks of gestation. The postpartum course was uneventful and the newborn was discharged to home care 63 days after delivery.

**Conclusions:**

Fetal expulsion into the abdominal cavity after silent uterine rupture of the scarred uterus may be accompanied by minimal symptomatology making early diagnosis difficult. This rare complication must be considered in the differential diagnosis in women after major uterine surgery. In selected cases and under conditions of intensive maternal and fetal monitoring, conservative management may be chosen to reduce the risks associated with prematurity.

## Background

Uterine rupture is one of the most serious complications in pregnancy. Most cases involve pregnancies with the presence of uterine scar and occur peripartum. In cases with heavy bleeding, the life of the mother and fetus is at immediate risk [1, 2]. The solution is to perform an acute cesarean section. Uterine ruptures are usually divided in two groups - complete and incomplete (dehiscence). Incomplete rupture of the uterus is mostly defined as a process of gradual or complete rupture of the myometrium when the serosa and amniotic sac are intact and the patient is usually asymptomatic. Complete uterine rupture is used for situations of complete interruption of uterine wall continuity along with severe clinical manifestations (intra-abdominal bleeding, tachycardia, abdominal pain) [3, 4]. Antenatal silent uterine rupture is characterized by a complete uterine rupture with minimal clinical manifestations significantly hindering early diagnosis. In these cases, partial or complete expulsion of the fetus into the abdominal cavity may occur. According to the literature, the reported maternal mortality in advanced abdominal pregnancy ranges from 0.5 to 18%. The risk of fetal death is reported between 40 and 95% [5–7]. Secondary abdominal pregnancy resulting from fetal expulsion by uterine rupture in the second or third trimester is one of the rare complications. The high rate of complications is the reason for pregnancy termination, usually immediately after diagnosis [8].

We describe a unique case of secondary abdominal pregnancy after scar dehiscence with subsequent silent rupture of the thinned part of the uterine wall with the choice of a conservative approach and prolongation of pregnancy by three weeks, followed by elective surgery with a good outcome for both mother and fetus.

## Case report

A 43-year-old tercigravida/primipara was referred to the Obstetrics and Gynaecology Department of Masaryk University and Faculty Hospital Brno due to the oligohydramnios detected in the 24th week of pregnancy. She had a history of right-sided salpingectomy for ectopic pregnancy 5 years ago, and laparotomic myomectomy of a 6 cm-large transmural myoma from the posterior wall without the interference of the uterine cavity with plastic suture of the uterine wall and resection of part of the omentum with endometriosis lesions one year later. Spontaneous pregnancy occurred two years after the myomectomy and was ended by an elective cesarean section performed by Pfannenstiel incision at term due to a 7 cm-large myoma in the lower uterine segment forming a birth obstruction. The description of the scar after the previous myomectomy is missing.

The current spontaneous pregnancy appeared two years after the previous cesarean section. In early pregnancy, the patient was treated in our emergency department for cramping pain around the umbilicus and lower abdomen. One gestational sac without embryonic structures in the uterus and a 6 cm-large intramural myoma on the anterior uterine wall were described. In the 13th week of gestation, the patient was referred to our department due to the finding of cystic resistance in the small pelvis. The ultrasound described a viable fetus in the uterine cavity, a 7 cm-large intramural myoma in the right uterine edge, a placenta located on the posterior uterine wall, and a septated cyst 9 × 7 cm above the uterus. The patient was scheduled for an expert ultrasound examination to specify the cyst, but she failed to come for this scan. First and second-trimester screening for fetal malformations was performed in a prenatal diagnostics center, with the description of intrauterine pregnancy without any pathology, apart from a 6 cm-large unilocular cyst on the left side without abnormal vascularization during the first-trimester screening. The last ultrasound examination was the second-trimester scan at 22 + 0 weeks of gestation.

The patient retrospectively described repeated episodes of intense cramp-like abdominal and back pain at 20 and 22 weeks of pregnancy. However, she did not show up for examination with these complaints, as they were transient and lasted only a few hours.

**Fig. 1:**
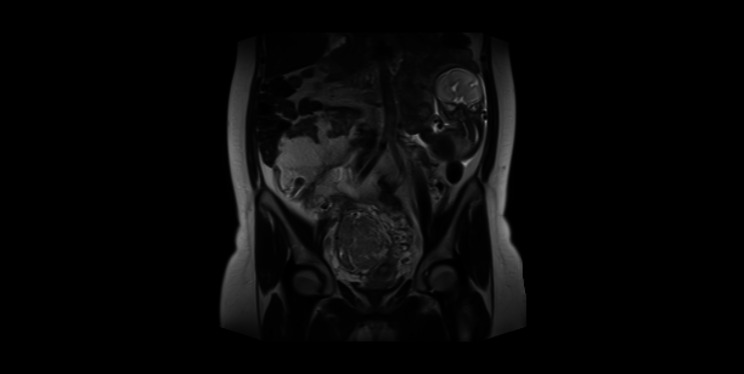
MRI examination at 25 + 0 weeks of gestation. The fetus is localized in the amniotic sac below the level of the spleen

She was reffered to our department at 24 weeks and six days due to the finding of oligohydramnios. The uterus was described without any findings of fetal structures. Eutrophic viable fetus presented freely in the amniotic sac in the left subcostal region, normal amount of amniotic fluid, placenta on the posterior uterine wall, 7 cm-large intramural myoma in the region of the lower uterine segment, normal doppler measurement in the umbilical artery. Hospital admission was indicated, fetal lung maturation was initiated, and MRI (Magnetic Resonance Imaging) was indicated to verify these findings. According to MRI, a defect of the uterine wall of 6 cm in the area of the right uterine horn and right uterine edge with eversion of the uterine wall adjacent to the placenta was described. The border of the myometrium and placental tissue could not be reliably differentiated, and suspicion of abnormal placental invasion into the uterine wall arose. The fetus was localized in the amniotic sac below the level of the spleen, with no evidence of free fluid in the abdominal cavity (see Fig. 1). Throughout the hospitalization, the patient was free of subjective complaints.

A detailed consultation with the patient in the presence of an obstetrician, neonatologist, and gynaecologic oncologist surgeon followed. Two possible procedures were proposed. First option - delivery immediately after completion of antenatal corticosteroids by elective cesarean section with possible hysterectomy due to the rupture of the uterus and morbidly adherent placenta suspicion. This option minimizes the risk to the mother but is burdened with an uncertain prognosis for the newborn due to severe prematurity at 24 weeks of pregnancy. The second option is an attempt to reach the completed 28th week of pregnancy to reduce morbidity for the fetus due to severe prematurity, with intensive monitoring of the maternal and fetal well-being followed by a planned cesarean section with subsequent hysterectomy for the same reason. Still, it comes with the risk of sudden complications, especially intra-abdominal bleeding with the need for acute surgery, endangering the life of both mother and fetus. The parents chose the second option – conservative management in an attempt of extending the pregnancy.

Hospitalization in the ICU (intensive care unit) continued. Central venous access was established, blood transfusions were permanently available, and the services of a gynecologic oncologist, available 24 h a day, were scheduled. Fetal ultrasound checks daily, fetal heart rate checks twice daily, laboratory results updated twice weekly, patient support by a psychologist, and nutritional support with protein supplementation. The whole course of hospitalization was without serious complications.

**Fig. 2:**
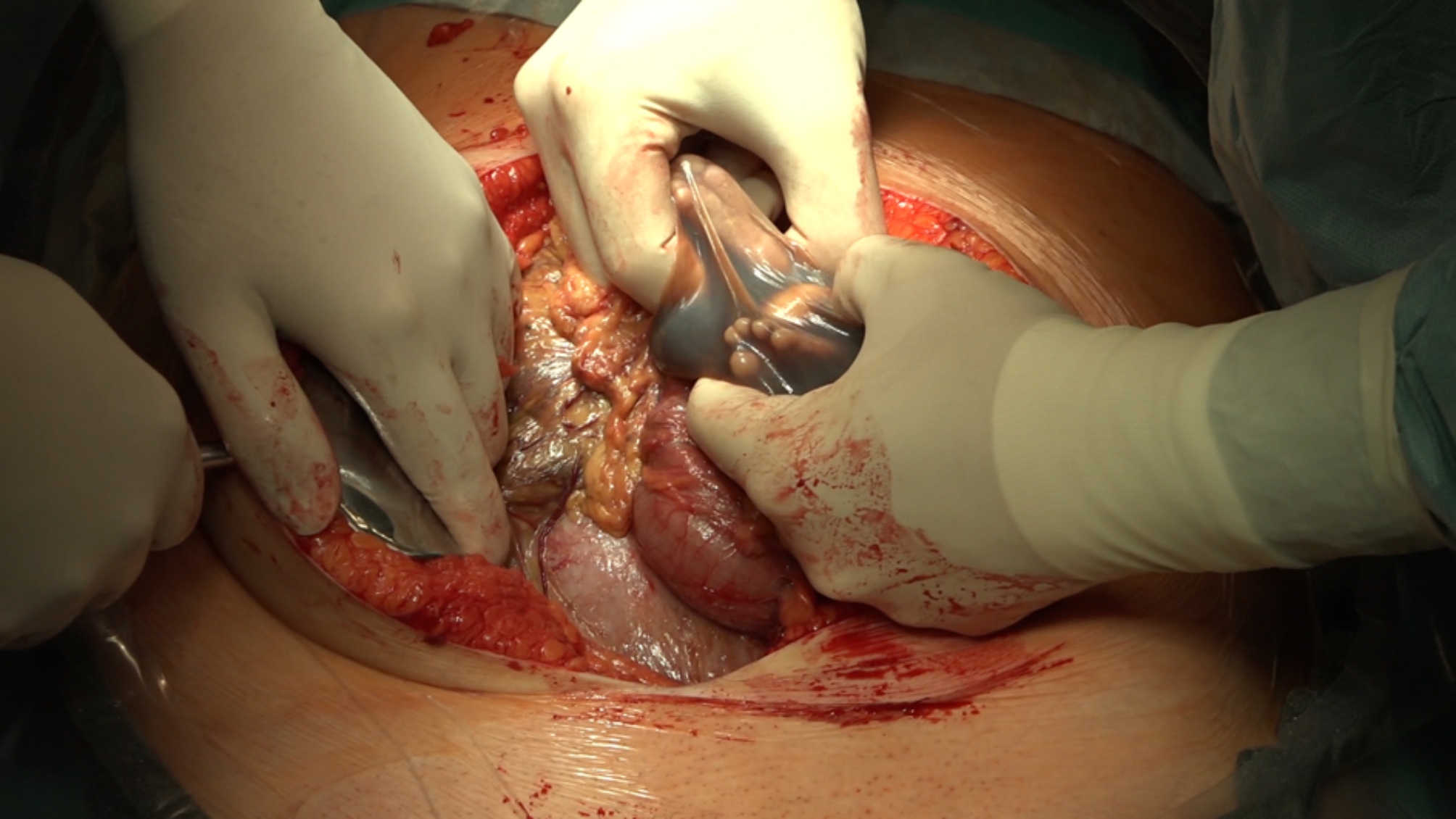
Surgical procedure at 28 + 0 weeks of gestation. The fetus lodged loosely in the left subcostal space and next to the left uterine edge in an intact amniotic sac between the intestinal loops

Pregnancy was ended electively at 28 weeks and 0 days of gestation. A cesarean section was performed from lower midline laparotomy. The fetus lodged in the left subcostal space and next to the left uterine edge in an intact amniotic sac between the intestinal loops (see Fig. 2). The fetus was easily handled, the amniotic fluid slightly stained, with old blood remnants. Baby girl born with birth weight 990 g, Apgar score 8-9-10, pH of umbilical artery 7.38.

During the surgical procedure, extensive uterine wall defect in the fundus and posterior uterine wall on the side of the previous myomectomy was observed. The smaller part of the placenta was located in the uterus and the larger part was prolapsed outside the uterus and firmly attached to the area of the uterine fundus and the edges of the uterine wall defect. Subsequently, the surgeon performed extensive adhesiolysis, gradually releasing the omentum and the bowel loops adhering to the area of the fundus and posterior uterine wall. Hysterectomy, partial omentectomy, appendectomy, and bilateral ureterolysis were added. This was a technically challenging procedure due to the extensive adhesive process in the small pelvis related to endometriosis and the localization of intramural myoma in the right uterine edge in the lower uterine segment. Total blood loss was 800 ml, and the patient was transferred to the intensive postoperative care unit after the procedure. The postoperative course was uneventful, and the patient was transferred to the standard ward on the 3rd day after surgery.

The baby girl was born vigorously without the need for resuscitation. Noninvasive respiratory support for mild respiratory distress syndrome was applied for 12 days. She spent 25 days in our Neonatal Intensive Care Unit (NICU). Empiric antibiotic therapy was stopped after 3 days due to negative blood culture. After 5 days of parenteral nutrition, she was on full enteral feeding. A blood transfusion was given three times. Brain ultrasound was without abnormalities and cardiology examination showed normal anatomy and function. Breastfeeding was replaced by formula because of poor lactation. She was discharged after 63 days of hospitalization with a weight of 2210 g.

**Fig. 3:**
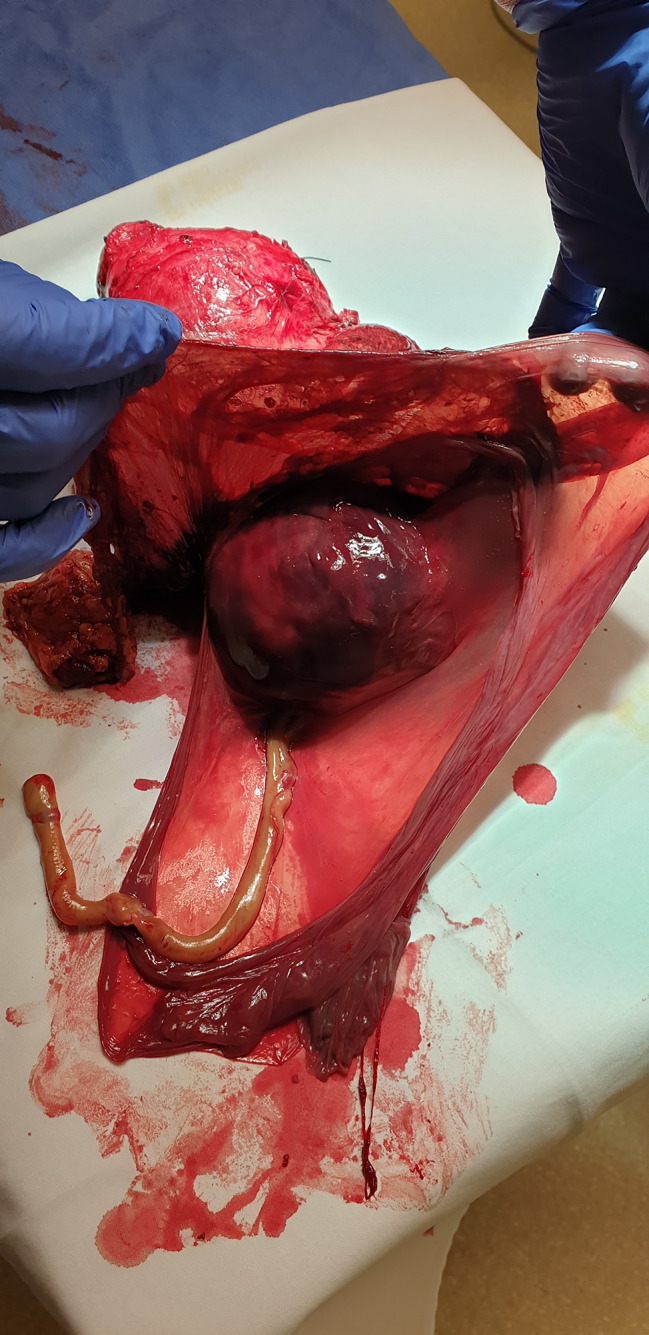
The whole uterus after hysterectomy, placenta, and amniotic sac

The pathologist described only one umbilical artery, velamentous insertion of the umbilical cord, and insertion of the placenta into the muscular layer of the uterine wall and partly in the serous part of the uterine wall around the edges of the uterine defect (see Fig. 3). The suspicion on the morbidly adherent placenta was confirmed.

## Discussion

The risk of uterine rupture in patients with uterine scar during pregnancy and delivery is around 1/100, while the risk in the previously unoperated uterus is reported to be less than 1/10.000 [9–11]. In acute complete uterine wall rupture, there is a risk of fetal death from asphyxia due to placental abruption and severe bleeding [1]. Silent uterine rupture during pregnancy cannot always be diagnosed in time due to the absence of typical clinical signs. Naim et al. described a uterine rupture with subsequent fetal expulsion into the abdominal cavity, diagnosed at 36 weeks of gestation. The author concludes that the uterine rupture and fetal expulsion probably occurred at around 30 weeks; however, due to non-specific symptoms, this complication was not picked up. The pregnancy was ended immediately after diagnosis, and a healthy newborn was delivered [12].

The choice of conservative management for uterine rupture is rarely described. Only very few case reports are available in the literature. In this context, cases of uterotomy dehiscence with subsequent protrusion of amniotic membranes are most frequently described. Iemura et al. described a case of uterotomy dehiscence after myomectomy with protrusion of the amniotic sac diagnosed at 18 weeks of gestation with the successful extension of pregnancy to 30 weeks [13]. Hamar et al. describe a case with conservative management of uterotomy dehiscence diagnosed in the lower uterine segment at 20 weeks with subsequent termination of pregnancy by cesarean section at 31 weeks due to the detection of an abnormal cardiotocographic recording [14]. Oyelese et al. published a case of uterine fundus dehiscence with fetal membranes herniation and transient fetal limb and umbilical cord protrusion through the defect, diagnosed at 23 weeks. The pregnancy was electively terminated by cesarean section at 33 weeks due to abnormal fetal heart rate (FHR), and a healthy baby was born [2]. In 1982, Cotton et al. described a case of partial fetal expulsion into the abdominal cavity during uterine wall rupture in the region of the uterine fundus in a patient repeatedly examined for abdominal pain from the 20th week. Cesarean section was performed at 29 weeks, immediately after confirmation of the diagnosis [15]. Rabinowitz. et al. described a similar case with protrusion of the fetal membranes and fetal limb through a defect at 27 weeks of gestation. The pregnancy ended at 32 weeks of gestation due to spontaneous amniotic fluid leakage [10]. In the case we present, a complete expulsion of the fetus into the abdominal cavity was diagnosed. After consultation with the patient, a conservative procedure was chosen after considering all the risks. Pregnancy was successfully prolonged by more than three weeks.

Prediction of uterine rupture is practically impossible. A targeted ultrasound examination of the uterine wall at the site of a previous uterotomy can be offered. However, even this examination has limitations, as there are no clear cut-off values for uterine wall thickness that reliably predict uterine rupture [13, 15].

Ultrasound visualization of a complete uterine wall defect is difficult and depends on localization. In the case of previous surgery on the posterior uterine wall, the use of ultrasound is very limited [11, 13]. This situation occured in the presented case report, where it was not possible to reliably exclude a certain degree of dehiscence in the area of the posterior uterine wall before the 20th weeks of pregnancy.

In cases where findings are unclear and the status of the mother and fetus is stable, it is advantageous to perform MRI. In our case report, MRI confirmed the diagnosis and clarified the description of the uterine defect.

The primary symptoms of uterine rupture include severe sharp abdominal pain, usually followed by signs of the onset of shock due to the development of intra-abdominal hemorrhage [16]. The fetus usually develops acute hypoxia. Dehiscence, described as an incomplete disruption of the integrity of the uterine wall, is often asymptomatic and can, therefore, completely miss the diagnosis [17]. In cases of progressive dehiscence with subsequent partial or complete fetal expulsion, repeated episodes of sharp transient abdominal pain of a non-specific character are most frequently described [12, 15]. Similarly, subjective symptoms were described in our case report. The patient retrospectively described several episodes of sharp abdominal pain that first appeared around the 20th weeks and always resolved spontaneously after a few hours.

Most of the cases of clinically silent uterine rupture with subsequent partial or complete fetal expulsion described in the literature were managed by urgent termination of pregnancy. In many of these cases, the authors admit a variable length of time between the onset of the complication and the diagnosis. Given the significant effect of gestational age on the newborn prognosis and outcome, conservative management could be an option for selected cases diagnosed at low gestational weeks [13]. Conservative management is possible only in asymptomatic cases with good maternal and fetal health and with intensive maternal and fetal monitoring. The patient and the medical staff must be prepared to deal immediately with sudden complications, especially those associated with life-threatening intra-abdominal bleeding. The decision of whether to preserve the uterus depends on the clinical circumstances. The size of the defect and the insertion of the placenta are important factors.

The presented case report demonstrated that pregnancy with complete fetal expulsion into the abdominal cavity after silent uterine rupture can be successfully prolonged under specific conditions. The delivery was delayed by more than three weeks, and the risk of perinatal morbidity and mortality resulting from severe prematurity was significantly reduced.

To our knowledge, no similar case has been described in the literature.

## Conclusion

Fetal expulsion into the abdominal cavity after silent uterine rupture of the scarred uterus may be accompanied by minimal symptomatology making early diagnosis difficult. This rare complication must be considered in women after major uterine surgery. In selected cases and under conditions of intensive maternal and fetal monitoring, conservative management may be chosen to reduce the risks associated with prematurity assuming the good condition of the fetus.

## Data Availability

All data generated or analysed in the casereport are included in this published article.

## Abbreviations

MRI: Magnetic Resonance Imaging
ICU: Intensive Care Unit
NICU: Neonatal Intensive Care Unit
FHR: Fetal Heart Rate
