# Correction: Conservative management of complete fetal expulsion into the abdominal cavity after silent uterine rupture—case report

**DOI:** 10.1186/s12884-023-05853-6

**Published:** 2023-07-22

**Authors:** Lukas Hruban, Anna Jouzova, Petr Janku, Vit Weinberger, Dagmar Seidlova, Tomas Juren, Jan Senkyrik, Jana Kadlecova, Jitka Hausnerova, Eva Jandakova

**Affiliations:** 1 Department of Obstetrics and Gynecology, University Hospital Brno and Medical Faculty, Masaryk University, Jihlavská 20, Brno, 625 00 Czech Republic; 2 Department of Health Sciences, Faculty of Medicine, Masaryk University, Brno, Czech Republic; 3 Department of Anesthesiology and Intensive Care, University Hospital Brno and Medical Faculty, Masaryk University, Brno, Czech Republic; 4 Department of Neonatology, University Hospital Brno and Medical Faculty, Masaryk University, Brno, Czech Republic; 5 Department of Pediatric Radiology, University Hospital Brno and Medical Faculty, Masaryk University, Brno, Czech Republic; 6 Department of Pathology, University Hospital Brno and Medical Faculty, Masaryk University, Brno, Czech Republic

**Correction: BMC Pregnancy Childbirth 23, 500 (2023)**

**https://doi.org/10.1186/s12884-023-05812-1**

Following publication of the original article [1], the authors identified an error in the author names. The given name and family name were erroneously transposed.

The incorrect author names are: Hruban Lukas, Jouzova Anna, Janku Petr, Weinberger Vit, Seidlova Dagmar, Juren Tomas, Senkyrik Jan, Kadlecova Jana, Hausnerova Jitka, Jandakova Eva

The correct author names are: Lukas Hruban, Anna Jouzova, Petr Janku, Vit Weinberger, Dagmar Seidlova, Tomas Juren, Jan Senkyrik, Jana Kadlecova, Jitka Hausnerova, Eva Jandakova

The author group has been updated above and the original article has been corrected.
